# Hypothermic Machine Perfusion in Deceased Donor Kidney Transplantation: Provider and Transplant Center Practices and Perspectives

**DOI:** 10.1111/ctr.70637

**Published:** 2026-08-10

**Authors:** Beatrice P. Concepcion, Laura A. Binari, Vasanthi Balaraman, Angie Nishio‐Lucar, Anju Yadav, Ankita Patel, Kenneth J. Woodside, Krista L. Lentine, M. Lee Sanders, Oluwafisayo O. Adebiyi, Sabiha M. Hussain, Shikha Mehta, Vignesh Viswanathan, Xingxing S. Cheng, Ronald F. Parsons

**Affiliations:** ^1^ University of Chicago Chicago Illinois USA; ^2^ Vanderbilt University Medical Center Nashville Tennessee USA; ^3^ University of Tennessee Health Science Center Memphis Tennessee USA; ^4^ University of Virginia Charlottesville Virginia USA; ^5^ Johns Hopkins University Baltimore Maryland USA; ^6^ Recanati‐Miller Transplantation Institute The Icahn School of Medicine at Mount Sinai New York New York USA; ^7^ Sharing Hope South Carolina Charleston South Carolina USA; ^8^ SSM Health Saint Louis University Hospital Transplant Center St. Louis Missouri USA; ^9^ University of Iowa Health Care Iowa City Iowa USA; ^10^ Indiana University School of Medicine Indianapolis Indiana USA; ^11^ Penn Transplant Institute Perelman School of Medicine at the University of Pennsylvania Philadelphia Pennsylvania USA; ^12^ University of Alabama Birmingham Alabama USA; ^13^ Stanford University Transplant Center Palo Alto California USA

**Keywords:** cold ischemia time, deceased donor kidneys, delayed graft function, kidney transplantation, machine perfusion, organ procurement

## Abstract

**Background:**

Hypothermic machine perfusion (HMP), an organ preservation technique used in deceased donor kidney transplantation, improves early outcomes compared to traditional static cold storage while also providing a loosely validated organ viability assessment.

**Methods:**

We designed a survey to better understand the practices, perceptions, and barriers to utilization of HMP. Surgeons, nephrologists, and coordinators were invited to participate in the survey via email.

**Results:**

Respondents (90% transplant surgeons) from 88 transplant centers which utilize HMP (35% of active kidney transplant centers) participated, representing 63% of total transplant volume in 2024. Respondents strongly agreed that HMP is helpful in kidney selection (92%), reduces delayed graft function (90%), and allows for scheduling flexibility (91%). A majority (74%) answered that the Organ Procurement Organization (OPO exclusively pumps kidneys. The most cited barrier to HMP use was OPO refusal (58%). Thresholds for resistance and flow varied widely with over 2/3 of respondents citing a specified acceptable cut‐off. Most respondents answered “sometimes” (60%) or “yes” (20%) that they would decline a kidney if HMP could not be accomplished for a kidney they wished to be pumped. Perceived pitfalls were underutilization of kidneys due to incorrect or inaccurate perfusion parameters, logistical challenges and costs of pumping, inexperience and technical difficulties leading to organ or vasculature injury, and lack of standardization in practice.

**Conclusions:**

HMP is used as a tool to assess organ viability and to overcome logistical hurdles by improving flexibility in operative timing. OPOs play a critical role in the ability to perform HMP.

**Abbreviations**
AST: American Society of Transplantation
CIT: cold ischemic time
DCD: donation after cardiac death
DDKT: Deceased donor kidney transplant
DGF: Delayed graft function
HMP: Hypothermic machine perfusion
KDPI: Kidney donor profile index
OPO: Organ procurement organization
OPTN: Organ procurement and transplantation network
OR: operating room
REDCap: Research Electronic Data Capture
US: United States

## 1 Introduction

Kidney transplantation is the treatment of choice for patients with advanced kidney disease who are suitable candidates. In the United States (US), approximately 28 000 kidney transplants were performed in 2024, of which approximately 77% were from deceased donors [1]. Deceased donor kidney transplantation (DDKT) confers a survival benefit for patients, but a major unwanted immediate complication is delayed graft function (DGF) [2]. DGF is defined as the requirement for dialysis within 7 days of transplant and can persist for up to 1–3 months posttransplant [3]. DGF is associated with longer hospital length of stay, higher readmission rates, higher total costs of care, increased risk of allograft rejection, and more intensive out‐of‐hospital care coordination [3, 4, 5]. The DGF rate after DDKT in the US has increased over the past decade and was 26% in 2023 [6]. Increasing rates of DGF are attributable to the increasing use of kidneys from donors after cardiac death (DCD), donors with acute kidney injury, and older donors in recent years, along with broader geographic organ sharing [6].

Non‐oxygenated hypothermic machine perfusion (HMP), also commonly referred to as “pumping”, is an organ preservation technique wherein fluid is perfused through the deceased donor kidney with the aim of reducing ischemia‐reperfusion injury [7]. Compared to static cold storage, HMP has been associated with reduced rates of DGF and superior allograft survival and potentially lower costs [8, 9, 10]. HMP also enables an assessment of the viability of a kidney after procurement based on pump parameters such as flow and resistance [11].

In August 2025, 72% of transplanted deceased donor kidneys in the US were preserved with HMP compared to 50% in 2020 [12]. Despite the increased utilization of HMP, the provider‐ and center‐level practices surrounding its use are largely unknown. Therefore, there is a need to improve understanding of the provider and transplant center practices and perspectives on HMP to optimize its utilization. The objectives of this study are to: (1) describe HMP practices and logistical considerations at a transplant center and provider level, (2) identify barriers in the utilization of HMP, and (3) describe provider perceptions regarding the role of HMP in DDKT.

## 2 Materials and Methods

### 2.1 Survey Design

The survey instrument was developed by the study authors with additional feedback obtained from the American Society of Transplantation (AST) Education Committee. The survey questions were developed based on clinical experience, literature review, and study aims. The survey was built and administered through the Research Electronic Data Capture (REDCap) web‐based application at the University of Chicago.

The final survey consisted of 34 questions. Survey items included multiple‐choice, multiple select, and free‐text responses. Participants were asked to identify their role (surgeon, nephrologist, procurement coordinator, other) and transplant center. Participants were first asked to describe their center's practices and logistical considerations. Then they were queried, using a Likert scale format, on their perceptions regarding the benefits of pumping. Finally, open‐ended responses were sought regarding the perceived benefits and pitfalls of pumping.

This study was given an exempt status by the University of Chicago Biological Sciences Division/University of Chicago Medical Center Institutional Review Board (IRB24‐1341). The clinical and research activities reported are consistent with the principles of the Declaration of Istanbul, as outlined in the Declaration of Istanbul and Organ Trafficking and Transplant.

### 2.2 Participants and Survey Administration

Transplant surgeons, transplant nephrologists and transplant coordinators were invited to participate in the survey via email. A web‐based invitation with a link to the survey was sent to potential participants through the AST Kidney Pancreas Community of Practice (KPCOP) Hub and a one‐time email invitation was sent through the American Society of Transplant Surgeon's (ASTS) listserv. Email invitations to the survey were also sent through personal invitations to professionals. Email invitations to the survey were sent to potential participants on December 16, 2024. The survey end date was February 28, 2025.

Participants who did not utilize HMP at their center or who did not participate in organ offer acceptance decisions were asked to exit the survey and were excluded from the analysis. Surveys that had < 20% of questions answered were omitted. For the quantitative analysis, responses were analyzed with each transplant center represented once in the primary analysis. For centers with multiple respondents, the representative respondent for the center was selected based on a hierarchical algorithm taking into consideration the respondent's role in the transplant program: (1) transplant surgeon; (2) transplant nephrologist; (3) a respondent from the “others” category (transplant coordinator or administrator, etc.). If there were multiple respondents in the same role, the respondent with the highest percentage of questions answered was selected. If there were multiple respondents in the same role and with the same percentage of questions answered, the respondent who completed the survey first was selected. All available free‐text responses from respondents were included in the qualitative analysis. These inclusion criteria were specified as a priori.

### 2.3 Sensitivity Analysis

A sensitivity analysis was performed wherein all respondents who participated in organ acceptance decisions and answered > 20% of the survey were included. This analysis allowed for a transplant center to have more than one respondent. Respondents who did not specify a transplant center were also included in the analysis.

### 2.4 Statistical Analysis

Responses to each survey question were described by percentages and frequencies. For questions where participants were asked to “select all that apply”, the denominator for calculating percentages was the number of participants responding to that question. As part of the sensitivity analysis, interrater agreement on questions with nominal (yes/no) variables was assessed among respondents (at least 3) from the same transplant center using Fleiss’ kappa, which measures agreement among multiple raters while accounting for agreement by chance.

Respondents also commented on two open‐ended questions: (1) perceived benefits associated with HMP, and (2) perceived pitfalls associated with HMP. Two authors (BPC and LB) reviewed respondent comments to identify common and contrasting themes and patterns. We purposively selected exemplary responses to provide in‐depth insight on the qualitative findings of potential benefits and pitfalls of pumping. All analyses were performed using STATA SE version 17.0 (StataCorp, College Station, TX).

## 3 Results

### 3.1 Survey Participants

**FIGURE 1:**
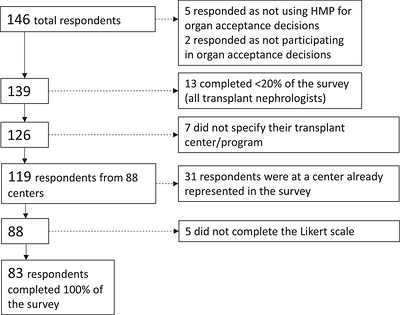
Survey participants.

There were 146 responses to the survey. Seven (5%) were excluded because HMP was not utilized at the respondent's center or they did not participate in organ acceptance decisions. Thirteen respondents (9%) were excluded after completing < 20% of the survey. Seven respondents (5%) did not specify their transplant center, and 31 (21%) respondents came from a center already represented in the study. Ultimately, 88 respondents from 88 distinct transplant centers were included in the primary analysis. Of these, 83 answered at least one question in the Likert scale portion of the survey. The inclusion/exclusion of survey participants is depicted in Figure 1. All of the Organ Procurement and Transplantation Network (OPTN) regions were represented in the analytical sample. Respondents represented 35% of US transplant centers and 63% of transplant volume in 2024.

Among the 88 respondents, the majority (90%) were transplant surgeons. Twenty‐three nephrologists started the survey but 13 were excluded due to answering < 20% of the questions. Ultimately, 9 (10%) transplant nephrologists were included in the primary analysis. Over half (54%) of respondents had greater than 10 years of transplant experience and 74% had greater than 5 years of experience with HMP.

### 3.2 Pumping Logistics

Most respondents (74%) reported that the Organ Procurement Organization (OPO) managing the donor, rather than the center, exclusively pumps kidneys for organs accepted at their center. Many centers did not own pumps (75%) nor had the requisite personnel (i.e. perfusionists) (86%) to perform it. Most (84%) local OPO's have a policy for pumping kidneys.

### 3.3 Provider Practices

More than two‐thirds of respondents agreed that the reasons HMP is requested include: to reduce DGF, to allow flexibility in operative timing, and to aid in organ selection (Table 1A). The deceased donor characteristics that respondents use as an indication to request HMP are listed in Table 1B. Over 90% of respondents selected DCD donors and long expected cold ischemia time (CIT) as characteristics to request HMP, whereas a minority selected donors with hypertension or vascular disease (33%) and donors with diabetes mellitus (22%).

**TABLE 1:** Provider practices.

**A. Reasons pumping is requested**	***N* = 88 (%)**
Reduce delayed graft function	77 (88)
Allow flexibility in operating room timing	67 (76)
Aid in organ selection	64 (73)
**B. Characteristics which determine if a kidney should be pumped**	***N* = 88 (%)**
Donation after cardiac death donor	80 (91)
Long expected cold ischemic time	80 (91)
Donor with acute kidney injury	73 (83)
Donor with kidney donor profile index > 85%	67 (76)
Kidneys from older donors, i.e. > 60 years regardless of DCD/DBD status	54 (61)
Multiorgan transplant	34 (39)
Donors with hypertension or vascular disease	29 (33)
Donors with diabetes mellitus	19 (22)
All kidneys are pumped regardless of donor characteristics	6 (7)

**FIGURE 2:**
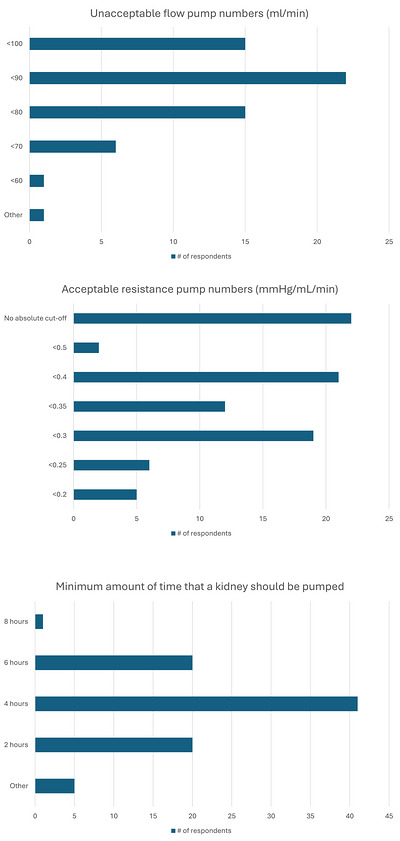
Provider practices (A) Unacceptable flow pump numbers, (B) Acceptable resistance pump numbers, (C) Minimum amount of time that a kidney should be pumped.

Criteria for acceptable resistance pump numbers, unacceptable flow pump numbers, and minimum time on the pump varied among respondents and are shown in Figures 2A–C. Seventy‐five percent of respondents specified an absolute cut‐off for resistance, while two‐thirds noted an absolute cut‐off for flow. Among those with a cut‐off, thresholds for resistance and flow varied widely but most considered < 0.4 mmHg/mL/min acceptable and < 80 mL/min unacceptable, respectively. Almost half of respondents believed that 4 h should be the minimum amount of time that a kidney should be perfused.

Nearly a third of respondents (31%) answered that they request HMP on > 75% of deceased donor kidney transplants offered to them, and majority (90%) responded that they turn down < 25% of kidneys after requesting that they be pumped. When asked if respondents would decline a kidney if HMP could not be accomplished for a kidney they wished to be pumped, most respondents answered “sometimes” (60%) or “Yes” (20%). Two‐thirds (64%) felt that both acceptable kidney biopsy results and pump parameters are needed to accept a kidney.

### 3.4 Provider Perceptions on Pumping

Table 2 lists the barriers to pumping as perceived by the respondents. The most cited barriers were OPO refusal (58%) and OPO prohibiting transport on a pump (30%). A minority cited high cost (26%) and lack of adequate personnel (23%) as barriers.

**TABLE 2:** Perceived barriers to pumping.

	*N* = 88 (%)
Organ Procurement Organization refusal to pump	51 (58)
Organ Procurement Organization prohibits transport on pump	26 (30)
High cost	23 (26)
Lack of adequate personnel	20 (23)
Other	12 (14)
Prolonging the cold ischemia time	7 (8)
Institutional resistance	2 (2)

**FIGURE 3:**
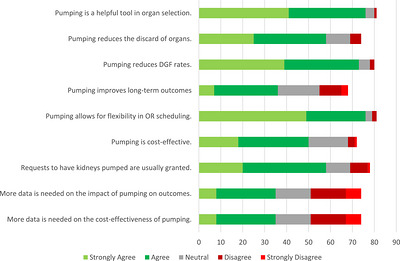
Likert scale responses to statements pertaining to the practice of pumping.

Eighty‐three respondents answered at least one question in the Likert scale with statements about the practice of pumping (Figure 3). Most respondents strongly agreed or agreed that pumping is a helpful tool in organ selection (92%), reduces DGF rates (90%), and allows for flexibility in operative scheduling (91%).

### 3.5 Sensitivity Analysis

There were 126 respondents representing at least 88 centers included in the sensitivity analysis, with 117 completing at least one question in the Likert scale (figure S1). Similar to the primary analysis, majority of respondents utilized pumping to reduce DGF (88%), to allow flexibility in OR timing (74%), and to aid in organ selection (72%). Likewise, responses to questions pertaining to characteristics which determine if a kidney should be pumped, acceptable/unacceptable pump parameters, and perceived barriers to pumping were similar to the primary analysis. A detailed comparison of the results of the sensitivity versus primary analyses are shown in tables S1–S3.

There were 7 transplant centers with at least 3 respondents included in the sensitivity analysis. Interrater agreement among respondents from the same center ranged from moderate agreement [Fleiss’ kappa of 0.49 (95% confidence interval 0.24, 0.84)] to almost perfect agreement [Fleiss’ kappa of 0.91 (95% confidence interval 0.77, 1.00]. Findings of the concordance analysis are summarized in table S4.

### 3.6 Thematic Categories of Beliefs on Benefits and Pitfalls Associated With Pumping

In open‐ended responses, respondents provided additional details and context on perceived benefits and pitfalls of pumping. Representative responses are listed in Table 3. Common themes of perceived benefits are that pumping provides more diagnostic information in organ acceptance decisions, helps with logistics such as operative timing, improves transplant outcomes, and increases kidney utilization. Perceived pitfalls are underutilization of kidneys due to incorrect or inaccurate pumping parameters, logistical challenges, costs, inexperience and technical difficulties leading to organ or vasculature injury, and lack of standardization in practice.

**TABLE 3:** Provider beliefs on benefits and pitfalls associated with pumping.

What do you believe are the benefits associated with pumping?	
Themes	Exemplary Quotes
More diagnostic information	*“Useful in AKI kidneys to determine whether DGF/PNF is likely”* *“1 of 3 parameters I used to determine whether to use kidney (along with biopsy and Cr)” “Evaluation of marginal organs and preservation for anticipated long CIT”* *“Allows for organ assessment and aid decision making when assessing an organ”* *“Decision making especially with high KDPI and complex background recipients. Very important for the assessment of DCDs and AKI kidneys”*
Logistical support	*“More flexibility in timing for marginal kidneys”* *“Safely increasing CIT for more OR flexibility in limited staffing situations”* *“Can accept multiple kidneys and facilitate sequential implantation”*
Improved outcomes and reduced delayed graft function	*“ I think pumping can help reduce DGF in higher risk kidneys and kidney selection in high‐risk donors”* *“…less DGF which simplifies post‐transplant management and may shorten hospital stay and costs. I think the increased costs of the pump are worth the improved short‐term outcome for the recipients”*
Increased utilization of kidneys	*“DCD and older age with WIT helps with organ selection”* *“…Utilization of organs from higher KDPI donors”* *“The opportunity to improve organ availability”* *“ Expands organ pool, specially when prolonged CIT is anticipated”*.
What do you believe are the pitfalls associated with pumping?	
Themes	Exemplary Quotes
Under‐utilization of kidneys due to incorrect or inaccurate pump parameters	*“Sometimes results are un‐reassuring and lead to turn downs that are a result of poor technical placement of the pump”* *“Placing too much emphasis on pump values when donor history is enough”* *“Malfunction of the machines/tubing leading to artificial poor pump numbers that dissuade centers from using”* *“Discard of potentially good organs when pump numbers are discordant with other organ information”*
Logistics and cost	*“Logistics of pump use may slow organ transfer and placement”* *“Logistics of putting on and monitoring pump, transportation and cost”* *“Limited amount of pumps available or perfusionists to put kidney on pump”*
Inexperience and technical difficulties leading to injury to organ or vasculature	*“Technical complications of placing the organ on the pump may occur. Pumps may fail and damage organs. ”* *“Depends on the technique of the person pumping and room for errors”* *“Getting them on the pump in a timely fashion, having appropriately trained personnel, incorrect cannulation that gives false numbers, vascular damage by untrained personnel”*
Lack of standardization	*“Absence of regional centers and individual OPOs creating criteria in the absence of transplant center input”* *“Lack of uniform practice”* *“Many technical variables that vary the consistency”* *“Some OPOs just keep turning up the pump pressure… to see if they can get adequate flow or resistance. There is no meaning or data associated with these high pump pressures to my knowledge”*

## 4 Discussion

In this national survey of transplant providers (90% of whom were transplant surgeons) regarding practices related to HMP in DDKT, we found that HMP is commonly utilized as a treatment modality *and* a tool for organ viability assessment. Almost three‐quarters of respondents utilize HMP to aid in organ selection. Respondents had variable practices as far as what was considered acceptable resistance, flow, and minimum time on the pump with a majority of respondents citing a specified cut‐off for resistance and flow.

The survey findings underscore that HMP is being utilized in current clinical practice as a tool to aid in kidney utilization decisions. Most respondents answered “sometimes” (60%) or “yes” (20%) when asked if they would decline a kidney if HMP could not be accomplished for a kidney they wished to be pumped. Notably, two‐thirds of the survey respondents used HMP parameters as criteria to accept or decline a kidney offer. While existing studies have demonstrated that high resistance and low flow are associated with DGF and graft loss [13, 14, 15, 16, 17, 18], the results are limited by low precision. In addition, studies have shown that some grafts with poor HMP parameters may ultimately have satisfactory outcomes. The available evidence suggests the need for individualized decision‐making and careful recipient selection [14, 18, 19, 20]. An analysis of data from the landmark prospective randomized controlled trial by Moers and colleague found that terminal renal resistance, that is renal resistance at the end of HMP, was an independent risk factor for DGF [odds ratio 21.12 (95% confidence interval 1.03, 435.0)] but the C‐statistic of the receiver operator characteristic curve was low (0.58) indicating poor predictive value [14]. Similarly, a study analyzing data from the randomized Consortium for Organ Preservation in Europe trial found that although terminal renal resistance predicted DGF [odds ratio 1.14, 95% confidence interval 1.009, 1.298], the C‐statistic of the receiver operating curve was also low [0.6 (95% confidence interval 0.55, 0.71)] [18]. In the former study, although the terminal resistance was associated with 1‐year graft failure, the confidence interval was very wide (hazard ratio 12.33 [95% confidence interval 1.11, 136.85]) indicating considerable uncertainty around the estimated effect. Taken together, these studies suggest that renal resistance should not be used as a standalone criteria for quality assessment and kidney discard due to insufficient precision. Several single center reports also highlight that despite poor pump parameters, these kidneys can still have reasonable outcomes. For example, a study comparing 91 kidneys with poor pump parameters (defined as terminal flow ≤ 80 mL/min and terminal resistance ≥ 0.4 mmHg/mL/min) with 598 kidneys with optimal pump parameters found that short‐term outcomes were comparable. Although the kidneys with poor pump parameters had inferior long‐term overall and death‐censored graft survival compared to the optimal pump parameter group, the authors noted that the outcomes of kidneys with poor pump parameters are comparable to outcomes that are to be expected of extended‐criteria donor kidneys indicating that these kidneys can still be utilized with careful recipient selection [18]. In our survey, nearly 70% of the respondents had various flow and resistance cut‐offs in the absence of clear guidelines or consensus [11, 14, 18, 20]. Just as with procurement biopsies [21], overreliance on “objective” HMP parameters can result in unnecessary discard of organs [11, 22, 23]. It is worth noting that in the trial by Moers and colleagues, applying an “optimal” cut‐off would not have avoided any of the cases of primary non‐function. In our survey's open‐ended responses, participants raised concerns of inaccurate HMP parameters leading to organ underutilization. The survey highlights that HMP is perceived as a tool by clinicians that can be utilized to make kidney utilization decisions despite available evidence not supporting this practice.

The survey also revealed that respondents utilize HMP to reduce the incidence of DGF. HMP has been shown to reduce DGF in multiple studies and meta‐analyses [8, 9, 24, 25, 26]; therefore, its increasing use in the current era for this purpose is not unexpected [12]. Kidneys from DCD donors and kidneys with long expected CIT, both known to be at risk for DGF, were cited as indications to pump a kidney by over 90% of respondents. The study by Moers et al. demonstrated that HMP was associated with a 43% reduction in the odds of DGF and conferred superior one‐year graft survival compared to static cold storage [8]. A more recent retrospective analysis of the United Network for Organ Sharing database found that kidneys on HMP for ≤ 24 h, compared to those on static cold storage, had less death‐censored graft loss [9]. This study also found that HMP was associated with a lower incidence of DGF at every level of CIT [9]. Following the implementation of the new circle‐based kidney allocation system in 2021, data have shown an increase in both CIT and the rate of DGF [6, 12]. This has led to a greater demand for HMP.

Utilizing HMP to allow flexibility in operative timing was another indication cited by most survey respondents. The increasing complexity of the transplant surgical procedure due to recipient co‐morbidities may lead to a preference for or necessity of performing surgeries during the day when more support is available, rather than in the middle of the night. Additionally, greater surgical volumes and limited operating room (OR) space may contribute to the need for flexibility in operative timing. In these settings, HMP expands a transplant program's operative capacity without compromising outcomes [9], an important consideration while evaluating the sustainability and cost‐effectiveness of HMP. HMP time to steady state parameters is rapid in most cases (1–2 h) and desire for more time (as respondents reported here) likely reflects logistical preferences rather than any significant change in pump parameters [16]. OPTN stakeholders should consider standardizing HMP time necessary for acceptance decisions while allowing kidneys to remain on the pump afterwards for logistical purposes.

The survey highlighted the major role of OPOs in mediating access to HMP. Majority of HMP is carried out by OPO's, and most centers do not own their own pumps nor have dedicated perfusionists. Respondents cited OPO refusal to pump and OPO refusal to transport kidneys on a pump as main barriers to pumping. A survey of 44 OPOs conducted in 2022 found that the top OPO indications for pumping kidneys were DCD kidneys (79%), transplant center request (70%), kidney donor profile index (KDPI) > 85% kidneys (66%), elevated creatinine (66%), and expected prolonged cold ischemic time (59%). The main OPO‐perceived barriers to pumping kidneys cited were lack of availability of staff (34%) and transportation (23%), whereas 45% cited no barriers [27]. Currently, national standards or guidance are absent for OPOs on when to offer HMP. This concern was voiced by respondents in open‐ended responses. Similar to the minimum procurement biopsy criteria implemented by the OPTN [28], it may be beneficial to develop standardized criteria for the utilization of HMP to ensure consistency and minimize friction at the time of the offer process. In addition, standards surrounding the transportation of pumps need to be developed to allow pumps to cross donor service area borders. Collaboration among adjacent OPOs could potentially address some of these logistical barriers. Finally, the cost of pumping is incorporated into the cost report and the standard acquisition charges of the OPO. While ultimately covered by the Medicare Cost Report and private payers through transplant centers, payment is indirect. In the current era of increasing deceased donor kidney utilization, HMP can potentially generate significant cost savings downstream by reducing DGF. However, the initial cost of HMP (borne by OPOs) and the cost of DGF (borne by transplant programs) are in different silos. Innovative ways to share the costs and savings related to HMP may help improve access to HMP.

Based on the survey findings, several measures are recommended to optimize the use of HMP in DDKT. First, standardized criteria for interpreting pump flow and resistance parameters should be developed to reduce variability and prevent unnecessary organ discard [11, 13, 14]. HMP data should be integrated with other clinical and histologic indicators rather than used in isolation, as some grafts with poor parameters still perform well [11, 19, 20]. The use of HMP remains appropriate for kidneys at high risk of DGF, such as those from DCD donors, donors with AKI, older age donors, donors with high KDPI, and kidneys with prolonged CIT, where evidence supports improved outcomes [8, 24]. To ensure equitable and consistent practice, national guidelines and OPO standards should be established to define indications, staffing, and transport protocols [27]. Finally, collaborative data sharing and logistical support across OPOs and transplant centers are essential to refine predictive models, improve decision‐making, and maximize organ utilization [6].

There are several strengths to our study. To our knowledge, this is the first study to describe provider perspectives on the real‐world use of HMP on a national scale, covering a broad range of topics including its indications, organ viability criteria, and perceived benefits, barriers, and pitfalls. Our study findings highlight the need for additional data to standardize clinical practice regarding acceptable HMP parameters and their thresholds, which our respondents demonstrated wide ranges of interpretation. Our findings can also serve as groundwork in developing guidelines for when and how to perform HMP to standardize practice across OPOs. Finally, our study identified barriers that can potentially be addressed, such as OPO challenges of transporting pumps across donor service area borders.

The study has several limitations. First, there is the possibility of selection bias inherent to an open survey. Second, the study had a relatively low response rate, although all OPTN regions were represented in the survey, and a substantial portion of total kidney transplant volume is represented nationally. Third, while there was only one respondent selected per transplant center, the respondents were providers who take organ offers and make organ acceptance decisions, thus representing real‐world practice and challenges. In addition, results of the sensitivity and primary analyses were similar and interrater agreement on responses that were analyzed were moderate to almost perfect. Fourth, the themes generated from the open‐ended responses lacked analysis via formal qualitative methods which could introduce bias to the results. Lastly, questions pertaining to acceptable flow were absolute and did not take into account kidney weight.

The results of the study should be viewed in the context that the survey questions and responses did not differentiate between continuous HMP, cold static storage combined with HMP (non‐continuous HMP), and end‐ischemic HMP. In addition, practices and perspectives regarding alternative perfusion types such as oxygenated HMP, normothermic machine perfusion and sub‐normothermic acellular machine perfusion performed at a kidney assessment and repair center were not queried as this was beyond the scope of the study and should be an area of future research [29].

## 5 Conclusions

In conclusion, this national study found that HMP is often used as a tool to assess organ viability with variation in perceived acceptable pump parameters despite evidence suggesting that HMP should not be used in utilization decisions. The study found that HMP is also utilized to overcome logistical hurdles, specifically by improving flexibility in operative timing. Finally, most respondents noted the critical role that OPOs play in the ability to perform HMP. With increased recovery and utilization of older and other higher‐risk kidneys prone to DGF, the demand for HMP is only expected to increase. Data contained herein can contribute to both clinical and financial planning to rationalize investment in the equipment and personnel for HMP, along with exploration of leveraging the advantages of pump portability in this era of increasing transportation logistic complexity and the corresponding increase in CIT during allocation and transportation.

## Disclosure

Beatrice P. Concepcion has received consulting fees and honoraria from IPSEN pharmaceuticals, Veloxis, Thermofisher and Novartis, is co‐chair of the National Kidney Foundation Health Equity Advisory Committee, and is a member of the American Society of Nephrology Public Policy and Advocacy Committee. Krista L. Lentine is a senior scientist of the Scientific Registry of Transplant Recipients (SRTR), scientific director of the SRTR Living Donor Collective, past chair of the American Society of Transplant (AST) Living Donor Community of Practice (COP), vice chair of the NLDAC Advisory Group, member of the American Society of Nephrology Transplant Committee, and member of the National Kidney Foundation Transplant Advisory Committee. Unrelated to this work, KLL receives consulting fees from CareDx and Maze Therapeutics, and speaker honoraria from Sanofi. Ronald F. Parsons receives research funding support from Natera, Transplant Genomics, ITB‐Med, Regeneron, Hansa Biopharma, is a member at large for the AST Living Donor Circle of Excellence, is chair of the American Society of Transplant Surgeons Cellular Transplantation Advisory Committee and is a member of the Pennsylvania Gift of Life Medical Advisory & Policy Board. The rest of the authors have nothing to disclose.

## Conflicts of Interest

The authors declare no conflicts of interest.

## Supporting information




**Supporting Information:** ctr70637‐sup‐0001‐TablesS1‐S2.docx

**Supporting Information:** ctr70637‐sup‐0002‐TableS3.docx

**Supporting Information:** ctr70637‐sup‐0003‐TableS4.docx

**Supporting Information:** ctr70637‐sup‐0004‐FigureS1.pptx

**Supporting Information:** ctr70637‐sup‐0005‐SuppMat.pdf

## Data Availability

Data is available from the authors upon request.
