# Supplementary material for: Hypothermic Machine Perfusion in Deceased Donor Kidney Transplantation: Provider and Transplant Center Practices and Perspectives
Source: Clin Transplant. 2026 Aug 10;40(8):e70637. doi: 10.1111/ctr.70637 (PMC13455452; doi:10.1111/ctr.70637)
Supplement: Supplementary file 1 [file CTR-40-e70637-s001.pdf]

# Impact of hypothermic machine perfusion (pumping) practices on kidney transplant access at the level of transplant centers

Dear Colleague,This is an invitation by the AST KPCOP Access Workgroup to participate in an online survey to understand provider and transplant center practices and perspectives on hypothermic machine perfusion ("pumping") as it relates to deceased donor kidney transplantation.Please share this link with colleagues at your institution dominantly involved in organ offers:https://redcap.link/pumpsurveyThe survey will take approximately 5-10 minutes to complete.By continuing to the next page, you consent to participate in the study, understanding the following:1. Participation is voluntary and uncompensated.2. Your responses will remain confidential and anonymous, although you will be asked which transplant center you are affiliated with.3. You can withdraw from the survey at any time.This study has been given an Exempt status by the University of Chicago Biological Sciences Division/University of Chicago Medical Center IRB (IRB24-1341). If you have any questions or concerns about the survey, please feel free to contact Dr. Beatrice Concepcion at 615-906-45473 or Beatrice.concepcion@bsd.uchicago.edu.

## INTRODUCTION

| | |
|---|---|
| Do you take donor call for deceased donor kidney offers at your center? | ◯ Yes<br>◯ No |
| What is your role? | ◯ Transplant surgeon<br>◯ Transplant nephrologist<br>◯ Transplant/procurement coordinator<br>◯ Other |
| Please specify role | __________________________________ |
| How long have you been in practice in the field of transplantation? | ◯ Less than 5 years<br>◯ 5-10 years<br>◯ Greater than 10 years |

What is your UNOS/OPTN center code?

◯ ALCH - Children's of Alabama
◯ ALUA - University of Alabama Hospital
◯ ALVA - Birmingham VA Medical Center
◯ ARCH - Arkansas Children's Hospital
◯ ARUA - UAMS Medical Center
◯ AZCH - Phoenix Children's Hospital
◯ AZGS - Banner-University Medical Center Phoenix
◯ AZMC - Mayo Clinic Hospital Arizona
◯ AZSJ - St. Joseph's Hospital and Medical Center
◯ AZUA - Banner University Medical Center-Tucson
◯ CASD - University of California San Diego Medical Center
◯ CAGH - Scripps Green Hospital
◯ CALL - Loma Linda University Medical Center
◯ CACL - Children's Hospital Los Angeles
◯ CACS - Cedars-Sinai Medical Center
◯ CAUC - University of California at Los Angeles Medical Center
◯ CAUH - Keck Hospital of USC
◯ CAIM - University of California Irvine Medical Center
◯ CASJ - Saint Joseph Hospital
◯ CAPC - Lucile Salter Packard Children's Hospital at Stanford
◯ CASM - University of California Davis Medical Center
◯ CASH - Sharp Memorial Hospital
◯ CACH - Rady Children's Hospital and Health Center
◯ CAPM - California Pacific Medical Center-Van Ness Campus
◯ CAMB - UCSF Medical Center at Mission Bay
◯ CASF - University of California San Francisco Medical Center
◯ CASU - Stanford Health Care
◯ CALA - Harbor UCLA Medical Center
◯ COCH - Children's Hospital Colorado
◯ COUC - University of Colorado Hospital/Health Science Center
◯ COPM - AdventHealth Porter
◯ COSL - Presbyterian/St Luke's Medical Center
◯ CTHH - Hartford Hospital
◯ CTYN - Yale New Haven Hospital
◯ DCCH - Children's National Medical Center
◯ DCGU - Medstar Georgetown Transplant Institute
◯ DCGW - George Washington University Hospital
◯ DCWH - Washington Hospital Center
◯ DCWR - Walter Reed National Military Medical Center at Bethesda
◯ DECC - Christiana Care Health Services
◯ DEAI - Nemours Children's Hospital Delaware
◯ FLHM - Halifax Health
◯ FLUF - UF Health Shands Hospital
◯ FLMR - Memorial Regional Hospital
◯ FLSL - Mayo Clinic Hospital Florida
◯ FLLM - Largo Medical Center
◯ FLJM - Jackson Memorial Hospital University of Miami School of Medicine
◯ FLFH - AdventHealth Orlando
◯ FLSH - Ascension Sacred Heart Pensacola
◯ FLTG - Tampa General Hospital
◯ FLCC - Cleveland Clinic Florida Weston
◯ GAEH - Children's Healthcare of Atlanta at Egleston
◯ GAEM - Emory University Hospital
◯ GAPH - Piedmont Hospital
◯ GAMC - Wellstar MCG Health, affiliated with Medical College of Georgia
◯ HIQM - The Queen's Medical Center
◯ IAIM - Iowa Methodist Medical Center
◯ IAIV - University of Iowa Hospitals and Clinics

Transplant Programs
◯ IAVA - The Iowa City VA Health Care System
◯ ILCM - Ann & Robert H. Lurie Children's Hospital of Chicago
◯ ILUC - University of Chicago Medical Center
◯ ILUI - University of Illinois Medical Center
◯ ILNM - Northwestern Memorial Hospital
◯ ILPL - Rush University Medical Center
◯ ILVA - Edward Hines Jr. Veterans Administration Medical Center
◯ ILLU - Loyola University Medical Center
◯ ILCH - Advocate Christ Medical Center
◯ ILSF - OSF Saint Francis Medical Center
◯ ILMM - Springfield Memorial Hospital
◯ INIM - Indiana University Health
◯ INSV - Ascension St. Vincent Hospital
◯ KSUK - University of Kansas Hospital
◯ KYUK - University of Kentucky Medical Center
◯ KYJH - Jewish Hospital
◯ KYKC - Norton Children's Hospital
◯ LATU - Tulane Medical Center
◯ LACH - Children's Hospital
◯ LAOF - Ochsner Foundation Hospital
◯ LAWK - Willis-Knighton Medical Center
◯ MABI - Beth Israel Deaconess Medical Center
◯ MAMG - Massachusetts General Hospital
◯ MANM - Tufts Medical Center
◯ MAPB - Brigham and Women's Hospital
◯ MABU - Boston Medical Center
◯ MACH - Boston Children's Hospital
◯ MALC - Lahey Clinic Medical Center
◯ MABS - Baystate Medical Center
◯ MAUM - UMass Memorial Medical Center
◯ MDJH - Johns Hopkins Hospital
◯ MDUM - University of Maryland Medical System
◯ MEMC - Maine Medical Center
◯ MIUM - University of Michigan Medical Center
◯ MICH - Children's Hospital of Michigan
◯ MIHF - Henry Ford Hospital
◯ MISJ - Ascension St. John Hospital
◯ MISM - Mercy Health Saint Mary's
◯ MIDV - Helen DeVos Children's Hospital
◯ MIBH - William Beaumont Hospital
◯ MNAN - Abbott Northwestern Hospital
◯ MNHC - Hennepin County Medical Center
◯ MNUM - University of Minnesota Medical Center, Fairview
◯ MNMC - Mayo Clinic Hospital Minnesota
◯ MOUM - University of Missouri Hospital and Clinics
◯ MOCM - Children's Mercy Hospital
◯ MOLH - St Luke's Hospital of Kansas City
◯ MORH - Research Medical Center
◯ MOSL - SSM Health Saint Louis University Hospital
◯ MOBH - Barnes-Jewish Hospital
◯ MOCG - Cardinal Glennon Children's Hospital
◯ MOCH - St. Louis Children's Hospital at Washington University Medical Center
◯ MSUM - University of Mississippi Medical Center
◯ NCMH - University of North Carolina Hospitals
◯ NCCM - Carolinas Medical Center
◯ NCDU - Duke University Hospital
◯ NCEC - ECU Health Medical Center
◯ NCBG - Wake Forest Baptist Medical Center
◯ NDMC - Sanford Bismarck Medical Center
◯ NDSL - Sanford Medical Center Fargo
◯ NEUN - The Nebraska Medical Center
◯ NHDH - Mary Hitchcock Memorial Hospital
◯ NJLL - Virtua Our Lady of Lourdes Hospital
◯ NJHK - Hackensack University Medical Center
◯ NJSB - Saint Barnabas Medical Center
◯ NJRW - Robert Wood Johnson University Hospital

◯ NJBI - Newark Beth Israel Medical Center
◯ NMAQ - University Hospital, University of New Mexico Health Sciences Center
◯ NMPH - Presbyterian Hospital
◯ NVUM - University Medical Center of Southern Nevada
◯ NYAM - Albany Medical Center Hospital
◯ NYMA - Montefiore Medical Center
◯ NYVA - James J. Peters VA Medical Center
◯ NYDS - State University of New York, Downstate Medical Center
◯ NYEC - Erie County Medical Center
◯ NYNS - North Shore University Hospital/Northwell Health
◯ NYNY - New York-Presbyterian Hospital/Weill Cornell Medical Center
◯ NYUC - NYU Langone Health
◯ NYMS - Mount Sinai Medical Center
◯ NYCP - NY Presbyterian Hospital/Columbia Univ. Medical Center
◯ NYCC - Long Island Jewish Medical Center-Cohen Children's Medical Center
◯ NYFL - Strong Memorial Hospital, University of Rochester Medical Center
◯ NYSB - University Hospital of State University of New York at Stony Brook
◯ NYUM - State University of New York Upstate Medical University
◯ NYWC - Westchester Medical Center
◯ OHTC - The Christ Hospital
◯ OHUC - University of Cincinnati Medical Center
◯ OHCM - Children's Hospital Medical Center
◯ OHUH - University Hospitals of Cleveland
◯ OHCC - The Cleveland Clinic Foundation
◯ OHCH - Nationwide Children's Hospital
◯ OHOU - Ohio State University Medical Center
◯ OHCO - University of Toledo Medical Center
◯ OKBC - Integris Baptist Medical Center
◯ OKMD - OU Medical Center
◯ OKSJ - St John Medical Center
◯ ORGS - Legacy Good Samaritan Hospital and Medical Center
◯ ORUO - Oregon Health and Science University
◯ ORVA - VA Portland Health Care System
◯ PALV - Lehigh Valley Hospital
◯ PAGM - Geisinger Medical Center
◯ PAPH - UPMC Hamot
◯ PAHH - Pinnacle Health System at Harrisburg Hospital
◯ PAHE - Penn State Milton S Hershey Medical Center
◯ PACP - Children's Hospital of Philadelphia
◯ PAAE - Albert Einstein Medical Center
◯ PASC - St. Christopher's Hospital for Children
◯ PATJ - Thomas Jefferson University Hospital
◯ PATU - Temple University Hospital
◯ PAUP - Hospital of the University of Pennsylvania
◯ PAVA - VA Pittsburgh Healthcare System
◯ PAAG - Allegheny General Hospital
◯ PACH - UPMC Children's Hospital of Pittsburgh
◯ PAPT - University of Pittsburgh Medical Center
◯ PRSJ - Auxilio Mutuo Hospital
◯ RIRH - Rhode Island Hospital
◯ SCMU - Medical University of South Carolina
◯ SCPG - Prisma Health Greenville Memorial Hospital
◯ SCLA - MUSC Lancaster
◯ SDMK - Avera McKennan Hospital
◯ SDSV - Sanford Health/USD Medical Center
◯ TNEM - Erlanger Medical Center
◯ TNUK - University of Tennessee Medical Center at Knoxville
◯ TNLB - Le Bonheur Children's Medical Center
◯ TNMH - Methodist University Hospital

◯ TNST - Saint Thomas Hospital
◯ TNVU - Vanderbilt University Medical Center
◯ TXDL - Dell Children's Medical Center
◯ TXDM - North Austin Medical Center
◯ TXDS - Dell Seton Medical Center at The University of Texas at Austin
◯ TXDC - Driscoll Children's Hospital
◯ TXHD - Medical City Dallas Hospital
◯ TXCM - Children's Medical Center of Dallas
◯ TXMC - Methodist Dallas Medical Center
◯ TXPM - Parkland Health and Hospital System
◯ TXSP - UT Southwestern Medical Center/William P. Clements Jr. University Hospital
◯ TXTX - Baylor University Medical Center
◯ TXDR - Doctor's Hospital at Renaissance
◯ TXLP - Las Palmas Medical Center
◯ TXPL - Medical City Fort Worth
◯ TXFW - Texas Health Harris Methodist Fort Worth Hospital
◯ TXCF - Cook Children's Medical Center
◯ TXAS - Baylor Scott and White All Saints Medical Center-Fort Worth
◯ TXJS - University of Texas Medical Branch at Galveston
◯ TXMH - Houston Methodist Hospital
◯ TXVA - Michael E. DeBakey VA Medical Center
◯ TXHH - Memorial Hermann Hospital, University of Texas at Houston
◯ TXHI - CHI St. Luke's Health Baylor College of Medicine Medical Center
◯ TXTC - Texas Children's Hospital
◯ TXUC - University Children's Health
◯ TXHS - Methodist Specialty and Transplant Hospital
◯ TXBC - University Hospital, University of Texas Health Science Center
◯ TXSW - Scott and White Memorial Hospital
◯ UTLD - Intermountain Medical Center
◯ UTMC - University of Utah Medical Center
◯ UTPC - Primary Children's Hospital
◯ VAUV - University of Virginia Health Sciences Center
◯ VAFH - Inova Fairfax Hospital
◯ VACH - Children's Hospital of the King's Daughters
◯ VANG - Sentara Norfolk General Hospital
◯ VAHD - Henrico Doctors' Hospital
◯ VAMC - VCU Health System Authority, VCUMC
◯ VTMC - The University of Vermont Medical Center
◯ WACH - Seattle Children's Hospital
◯ WASM - Swedish Medical Center
◯ WAUW - University of Washington Medical Center
◯ WAVM - Virginia Mason Medical Center
◯ WASH - Providence Sacred Heart Medical Center & Children's Hospital
◯ WIUW - University of Wisconsin Hospital and Clinics
◯ WICH - Children's Hospital of Wisconsin
◯ WISE - Froedtert Memorial Lutheran Hospital
◯ WISL - Aurora St. Luke's Medical Center
◯ WVCA - Charleston Area Medical Center
◯ WVWU - West Virginia University Hospitals Inc.
◯ Other

---

Please specify your UNOS/OPTN center code or center name

__________________________________

## PRACTICES AND LOGISTICS

Does your center utilize hypothermic machine perfusion (pumping) data to assist with organ acceptance decisions?

◯ Yes
◯ No

If not, why not?

__________________________________

Thank you for your time and participation. You may now exit the survey and close the browser.

__________________________________

For approximately what percentage of deceased donor kidney transplant offers do you request pumping?

◯ < 25%
◯ 25-50%
◯ 51-75%
◯ >75%

## PRACTICES AND LOGISTICS (continued)

Do you participate in organ offer acceptance decisions?

◯ Yes
◯ No

If not, why not?

__________________________________

Thank you for your time and participation. You may now exit the survey and close the browser.

__________________________________

## PRACTICES AND LOGISTICS (continued)

How long have you had experience with pumping?

- ◯ < 1 year
- ◯ 1-5 years
- ◯ >5 years

Who pumps the kidneys for those accepted at your center?

- ◯ OPO exclusively
- ◯ Transplant center exclusively
- ◯ Either the OPO or transplant center
- ◯ Third-party perfusion service
- ◯ Other
- ◯ I don't know

Please specify

__________________________________

Does your transplant center own a pump?

- ◯ Yes
- ◯ No
- ◯ I don't know

Does your transplant center have personnel (perfusionists) to pump kidneys?

- ◯ Yes
- ◯ No
- ◯ I don't know

Approximately what percentage of deceased donor kidneys that you accept arrive on a pump wherein pumping was not requested by you?

- ◯ < 25%
- ◯ 25-50%
- ◯ 51-75%
- ◯ >75%

Does your local OPO have a policy to pump kidneys?

- ◯ Yes
- ◯ No
- ◯ I don't know

What is/are the reason/s you request that a kidney be pumped? (select all that apply)

- ☐ To aid in organ selection
- ☐ To reduce delayed graft function (need for dialysis in the first 7 days posttransplant)
- ☐ To allow flexibility in OR timing
- ☐ Other

Please specify

__________________________________

Which characteristics determine if a kidney should be pumped? (select all that apply)

- ☐ DCD
- ☐ KDPI >85% donor
- ☐ Donors with AKI
- ☐ Long expected CIT
- ☐ Kidneys from older donors, i.e. >60 y, regardless of DCD/DBD status
- ☐ Donors with DM
- ☐ Donors with HTN or vascular disease on biopsy
- ☐ Multivisceral transplant
- ☐ All kidneys are pumped, regardless of donor characteristics
- ☐ Other

Please specify

__________________________________

| | |
|---|---|
| For approximately what percentage of kidneys do you turn down after requesting that they be pumped? | ◯ < 25%<br>◯ 25-50%<br>◯ 51-75%<br>◯ >75% |
| What is your cut-off for ACCEPTABLE resistance pump numbers? | ◯ < 0.15<br>◯ < 0.2<br>◯ < 0.25<br>◯ < 0.3<br>◯ < 0.35<br>◯ < 0.4<br>◯ < 0.5<br>◯ < 0.6<br>◯ I do not have an absolute cut-off<br>◯ Other |
| Please specify | __________________________________ |
| What is your cut-off for UNACCEPTABLE flow pump numbers? | ◯ < 100 ml/min<br>◯ < 90 ml/min<br>◯ < 80 ml/min<br>◯ < 70 ml/min<br>◯ < 60 ml/min<br>◯ I do not have an absolute cut-off<br>◯ Other |
| Please specify | __________________________________ |
| What is the minimum amount of time that you would want a kidney pumped? | ◯ 2 hours<br>◯ 4 hours<br>◯ 6 hours<br>◯ 8 hours<br>◯ Other |
| Please specify number of hours | __________________________________ |
| Will you turn down a kidney if pumping cannot be accomplished for a kidney you wish to be pumped? | ◯ Yes<br>◯ No<br>◯ Sometimes |
| In relation to procurement kidney biopsy results, which of the statement/s is/are true? (select all that apply) | ☐ Both biopsy and pump numbers should be acceptable for me to accept a kidney<br>☐ I will accept a kidney if pump numbers are acceptable regardless of biopsy results<br>☐ I will not accept a kidney if pump numbers are unacceptable regardless of biopsy results<br>☐ I will accept a kidney if biopsy results are acceptable irrespective of pump numbers. |
| Do you discuss the kidney acceptance decision with other colleagues at your center if the pump numbers are not reassuring? | ◯ Yes<br>◯ No |

## BARRIERS

What are the barriers to pumping? (select all that apply)

- ☐ High cost
- ☐ Lack of adequate personnel
- ☐ OPO refusal to pump
- ☐ Prolonging the CIT
- ☐ OPO transport is prohibited
- ☐ Institutional resistance
- ☐ Other

Please specify

__________________________________

**PERSPECTIVES**

**In the next section, please select from a scale of 1-5 how strongly you disagree or agree with the following statements.**

**1- strongly disagree**
**2- disagree**
**3- neutral**
**4- agree**
**5- strongly agree**

Pumping is a helpful tool in organ selection.

strongly disagree neutral strongly agree

*(Place a mark on the scale above)*

Pumping reduces the discard of organs.

strongly disagree neutral strongly agree

*(Place a mark on the scale above)*

Pumping reduces DGF rates.

strongly disagree neutral strongly agree

*(Place a mark on the scale above)*

Pumping improves long-term outcomes.

strongly disagree neutral strongly agree

*(Place a mark on the scale above)*

Pumping allows for flexibility in OR scheduling.

strongly disagree neutral strongly agree

*(Place a mark on the scale above)*

Pumping is cost-effective.

strongly disagree neutral strongly agree

*(Place a mark on the scale above)*

Requests to have kidneys pumped are usually granted.

strongly disagree neutral strongly agree

*(Place a mark on the scale above)*

More data is needed on the impact of pumping on outcomes.

strongly disagree neutral strongly agree

*(Place a mark on the scale above)*

More data is needed on the cost-effectiveness of pumping.

strongly disagree neutral strongly agree

*(Place a mark on the scale above)*

**PERSPECTIVES**

**Please answer the following questions with a free-text response.**

What do you believe are benefits associated with pumping? __________________________________

What do you believe are pitfalls associated with pumping? __________________________________
