# Supplementary material for: Hypothermic Machine Perfusion in Deceased Donor Kidney Transplantation: Provider and Transplant Center Practices and Perspectives
Source: Clin Transplant. 2026 Aug 10;40(8):e70637. doi: 10.1111/ctr.70637 (PMC13455452; doi:10.1111/ctr.70637)
Supplement: Supplementary file 2 [file CTR-40-e70637-s003.docx]

| **Likert Scale Statement** | Number of respondents | Strongly Agree (%) | Agree (%) | Neutral (%) | Disagree (%) | Strongly Disagree (%) |
| --- | --- | --- | --- | --- | --- | --- |
| More data is needed on the cost-effectiveness of pumping. | **97** | **15 (16)** | **38 (39)** | **20 (21)** | **14 (14)** | **10 (10)** |
|  | *69* | *10 (14)* | *26 (38)* | *15 (22)* | *11 (16)* | *7 (10)* |
| More data is needed on the impact of pumping on outcomes. | **102** | **12 (12)** | **38 (37)** | **23 (23)** | **21 (21)** | **8 (8)** |
|  | *74* | *8 (11)* | *27 (36)* | *16 (22)* | *16 (22)* | *7 (9)* |
| Requests to have kidneys pumped are usually granted. | **108** | **22 (20)** | **56 (52)** | **19 (18)** | **9 (10)** | **2 (2)** |
|  | *78* | *20 (26)* | *38 (49)* | *11 (14)* | *8 (10)* | *1 (1)* |
| Pumping is cost-effective. | **98** | **21 (21)** | **40 (41)** | **32 (33)** | **4 (5)** | **1 (1)** |
|  | *73* | *18 (25)* | *32 (44)* | *19 (26)* | *3 (4)* | *1 (1)* |
| Pumping allows for flexibility in OR scheduling. | **111** | **63 (57)** | **39 (35)** | **6 (5)** | **3 (3)** | **0 (0)** |
|  | *81* | *49 (60)* | *27 (33)* | *3 (4)* | *2 (3)* | *0 (0)* |
| Pumping improves long-term outcomes. | **94** | **11 (12)** | **40 (43)** | **30 (32)** | **10 (11)** | **3 (3)** |
|  | *68* | *7 (10)* | *29 (43)* | *19 (28)* | *10 (15)* | *3 (4)* |
| Pumping reduces DGF rates. | **110** | **48 (44)** | **53 (48)** | **7 (6)** | **2 (2)** | **0 (0)** |
|  | *80* | *39 (49)* | *34 (42)* | *5 (6)* | *2 (2)* | *0 (0)* |
| Pumping reduces the discard of organs. | **105** | **31 (29)** | **49 (47)** | **18 (17)** | **7 (7)** | **0 (0)** |
|  | *74* | *25 (34)* | *33 (45)* | *11 (15)* | *5 (7)* | *0 (0)* |
| Pumping is a helpful tool in organ selection. | **114** | **50 (44)** | **56 (49)** | **7 (6)** | **1 (1)** | **0 (0)** |
|  | *81* | *41 (51)* | *35 (43)* | *4 (5)* | *1 (1)* | *0 (0)* |

**Supplementary Table 3.** Likert Scale responses comparing all respondents (in **bold**) with representative respondent per center (in *italics*).

Abbreviations: DGF, delayed graft function; OR, operating room
