# Supplementary material for: Hypothermic Machine Perfusion in Deceased Donor Kidney Transplantation: Provider and Transplant Center Practices and Perspectives
Source: Clin Transplant. 2026 Aug 10;40(8):e70637. doi: 10.1111/ctr.70637 (PMC13455452; doi:10.1111/ctr.70637)
Supplement: Supplementary file 3 [file CTR-40-e70637-s004.docx]

**Supplementary Table 1. Provider practices comparing all respondents (N=121-126) with representative respondent per center (N=87-88)**

| **A. Reasons pumping is requested** | **N=88 (%)** | **N=126 (%)** |
| --- | --- | --- |
| Reduce delayed graft function | 77 (88) | 111 (88) |
| Allow flexibility in operating room timing | 67 (76) | 93 (74) |
| Aid in organ selection | 64 (73) | 91 (72) |
| **B. Characteristics which determine if a kidney should be pumped** | **N=88 (%)** | **N=126 (%)** |
| Donation after cardiac death donor | 80 (91) | 112 (89) |
| Long expected cold ischemic time | 80 (91) | 112 (89) |
| Donor with acute kidney injury | 73 (83) | 102 (81) |
| Donor with kidney donor profile index >85% | 67 (76) | 95 (75) |
| Kidneys from older donors, i.e. >60 years regardless of DCD/DBD status | 54 (61) | 78 (62) |
| Multiorgan transplant | 34 (39) | 49 (39) |
| Donors with hypertension or vascular disease | 29 (33) | 46 (37) |
| Donors with diabetes mellitus | 19 (22) | 29 (23) |
| All kidneys are pumped regardless of donor characteristics | 6 (7) | 12 (10) |
| **C. Criteria of acceptable pumping parameters** | **N=87 (%)** | **N=121 (%)** |
| ACCEPTABLE resistance pump numbers (mmHg/mL/min)   - <0.2 - <0.25 - <0.3 - <0.35 - <0.4 - <0.5 - No absolute cut-off | 5 (6)  6 (7)  19 (22)  12 (14)  21 (24)  2 (2)  22 (25) | 7 (6)  7 (6)  23 (19)  20 (17)  30 (25)  4 (3)  29 (24) |
| UNACCEPTABLE flow pump numbers (mL/min)   - <100 - <90 - <80 - <70 - <60 - No absolute cut-off - Other | 15 (17)  22 (25)  15 (17)  6 (7)  1 (1)  27 (31)  1 (1) | 18 (15)  27 (22)  23 (19)  9 (7)  7 (6)  36 (30)  1 (1) |
| Minimum amount of time that kidney should be pumped   - 2 hours - 4 hours - 6 hours - 8 hours - Other | 20 (23)  41 (47)  20 (23)  1 (1)  5 (6) | 27 (22)  63 (53)  24 (20)  2 (2)  45 (4) |

**Supplementary Table 2. Perceived barriers to pumping comparing all respondents (N=146) with representative respondent per center (N=88)**

|  | **N=88 (%)** | **N=126 (%)** |
| --- | --- | --- |
| Organ Procurement Organization refusal to pump | 51 (58) | 77 (61) |
| Organ Procurement Organization prohibits transport on pump | 26 (30) | 40 (32) |
| High cost | 23 (26) | 30 (24) |
| Lack of adequate personnel | 20 (23) | 34 (27) |
| Other | 12 (14) | 14 (11) |
| Prolonging the cold ischemia time | 7 (8) | 15 (12) |
| Institutional resistance | 2 (2) | 5 (4) |
