# Supplementary material for: Hypothermic Machine Perfusion in Deceased Donor Kidney Transplantation: Provider and Transplant Center Practices and Perspectives
Source: Clin Transplant. 2026 Aug 10;40(8):e70637. doi: 10.1111/ctr.70637 (PMC13455452; doi:10.1111/ctr.70637)
Supplement: Supplementary file 4 [file CTR-40-e70637-s005.docx]

**Supplementary Table 4. Concordance of survey responses among respondents from transplant centers with 3 or more study participants**

| Transplant center | 1 | 2 | 3 | 4 | 5 | 6 | 7 |
| --- | --- | --- | --- | --- | --- | --- | --- |
| Number of raters | 3 | 3 | 3 | 4 | 3 | 3 | 4 |
| Rating scale | Nominal (Yes/No) | Nominal (Yes/No) | Nominal (Yes/No) | Nominal (Yes/No) | Nominal (Yes/No) | Nominal (Yes/No) | Nominal (Yes/No) |
| Number of items rated | 29 | 29 | 29 | 29 | 29 | 29 | 29 |
| Overall percent agreement | 0.75 | 0.77 | 0.95 | 0.88 | 0.84 | 0.77 | 0.81 |
| Fleiss’ kappa | 0.49 | 0.54 | 0.91 | 0.77 | 0.66 | 0.54 | 0.63 |
| 95% confidence interval | 0.24,0.74 | 0.29, 0.79 | 0.77,1.00 | 0.57, 0.96 | 0.44, 0.89 | 0.29, 0.78 | 0.40, 0.86 |
| P value | <0.001 | <0.001 | <0.001 | <0.001 | <0.001 | <0.001 | <0.001 |
| Interpretation of agreement | Moderate agreement | Moderate agreement | Almost perfect agreement | Substantial agreement | Substantial agreement | Moderate agreement | Substantial agreement |
